# Evaluation of self-report screening measures in the detection of depressive and anxiety disorders among children and adolescents with systemic lupus erythematosus

**DOI:** 10.1177/09612033211018504

**Published:** 2021-06-02

**Authors:** Michelle Quilter, Linda Hiraki, Andrea M Knight, Julie Couture, Deborah Levy, Earl D Silverman, Ashley N Danguecan, Lawrence Ng, Daniela Dominguez, Katherine T Cost, Kate M Neufeld, Reva Schachter, Daphne J Korczak

**Affiliations:** 1 Department of Psychiatry, The Hospital for Sick Children, Toronto, Canada; 2 Department of Psychiatry, University of Toronto, Toronto, Canada; 3 Division of Rheumatology, The Hospital for Sick Children, Toronto, Canada; 4 Department of Paediatrics, University of Toronto, Toronto, Canada; 5 Division of Pediatric Rheumatology-Immunology, CHU Sainte-Justine, Montreal, Canada; 6 Department of Pediatrics, CHU Sainte-Justine, University of Montreal, Montreal, Canada; 7 Department of Psychology, The Hospital for Sick Children, Toronto, Canada

**Keywords:** Depression, anxiety, systemic lupus erythematosus, pediatric, screening

## Abstract

**Background:**

There are no validated screening measures for depressive or anxiety disorders in childhood Systemic Lupus Erythematosus (cSLE). We investigated cross-sectionally (1) the prevalence of depressive and anxiety disorder in cSLE. (2) the validity of the Centre for Epidemiologic Studies Depression Scale for Children (CES-DC) and the Screen for Childhood Anxiety and Related Disorders (SCARED) measures in identifyingthese disorders.

**Methods:**

Participants 8-18 years with cSLE/incipient cSLE completed CES-DC, SCARED, and Quality OfMy Life (QOML) measures. Parents completed the SCARED-Parent measure. Diagnosis was by gold-standard psychiatric interview and determined prevalence of psychiatric disorder. Receiver Operating Characteristics Area under the Curve (ROCAUC) evaluated screening measure diagnostic performance.

**Results:**

Ofseventy-two parent-child dyads, 56 interviews were completed. Mean screen scores were: CES-DC = 15 (range 1-49, SD 12), SCARED-C = 22 (range 2-61, SD 14), SCARED-P = 13 (range 0-36, SD 8). Depressive disorder screen positivity (CES-DC ≥ 15) was 35% (vs. prevalence 5%). Anxiety disorder screen positivity (SCARED ≥ 25) was 39% (vs. prevalence 16%). CES-DC ROCAUC = 0.98 and SCARED-C ROCAUC = 0.7 (cut-points 38 and 32 respectively).

**Conclusions:**

Diagnostic thresholds for depressive and anxiety disorderscreening measures are high for both CES-DC and SCARED-C in cSLE. Brief focused interview should follow to determine whether psychiatric evaluation is warranted.

## Introduction

Systemic Lupus Erythematosus (SLE) is a chronic, multisystem autoimmune disease which may present with greater disease severity in childhood. The propensity for neuropsychiatric SLE (NPSLE), as well as the common requirement for corticosteroid treatment for disease management, are risk factors for depressive disorder.^1,2^ Anxiety symptoms are frequently associated with chronic disease in children and may reach threshold for diagnosis of an anxiety disorder. Studies investigating the prevalence of depressive and anxiety symptoms in childhood-onset SLE (cSLE) are sparse, particularly with respect to anxiety.

Depressive and anxiety disorders in cSLE are known to impact negatively on disease self-management and treatment adherence which has been associated with increased healthcare utilization.^3,4^ Moreover, children and youth with SLE may be vulnerable to depressive and anxiety disorders for several reasons. The experience of a serious chronic health condition with associated treatment burden may lead to significant psychological distress, fearfulness and hopelessness for the future. Second, depressed mood may present as part of the symptom constellation of NPSLE. Third, corticosteroids administered for disease flares and NPSLE have a known association with adverse psychiatric effects, most commonly depressed mood.^5^ Joint pain and arthritis are also common disease manifestations, leading to chronic pain, a risk factor for depression and anxiety and poor quality of life.^6^ Finally, cSLE is known to present with more frequent episodes of disease activity and greater risk of hospitalization compared with adult-onset disease.^1,7^

A systematic review of the limited evidence available has shown wide-ranging prevalence rates of depressive and anxiety symptoms in cSLE, in part due to different screening measures, mixed disease samples and methodological limitations.^8^ Several studies investigating depressive and anxiety symptoms in cSLE have equated elevated screening measure scores with a diagnosis of depressive and anxiety disorders, despite the lack of a gold standard diagnostic psychiatric interview to confirm the disorder.^8^ Screening questionnaire measures are useful if they can accurately identify psychiatric disorder. However, the validity of screening measures in the detection of depressive and anxiety disorders among children with SLE has not been previously examined. Screening measures should be accurate and minimize the risk of false positive and false negative results. False positive screening results may lead patients to unnecessary consultations and/or prescription of psychotropic medication.^9^ In addition, falsely positive screening tests may lead children and families to misunderstand the etiology of their psychological distress, thereby resulting in other changes in behaviour which would otherwise not have occurred – a “nocebo effect”.^10,11^ False negatives represent a failure to identify psychiatric disorder, may reduce future healthcare seeking behaviour and may constitute a missed opportunity to address modifiable mental health risk factors.

Findings from studies of psychiatric symptoms in adult-onset SLE or in other chronic pediatric diseases cannot be simply extrapolated to make inferences about screening measure validity in the cSLE population. To our knowledge, there are no studies in cSLE which report on the prevalence of depressive and anxiety symptoms reaching diagnostic threshold for a Diagnostic and Statistical Manual of Mental Disorders fifth edition (DSM-5)^12^ psychiatric disorder. The aims of the present study were to examine the prevalence of depressive and anxiety disorders in cSLE, and to determine the diagnostic performance of commonly used screening measures to detect depressive and anxiety disorders against a validated gold-standard diagnostic psychiatric interview.

## Methods

### Participants

Participants were recruited from the paediatric SLE outpatient clinic at The Hospital for Sick Children between July 2017 and November 2019. Patients aged 8 to 18 years, and their families, were approached during routine clinic visits by a member of the research team. Patients were eligible to participate in the study if they met all three criteria: (1) diagnosed with SLE prior to 18 years of age, (2) diagnosed with SLE for at least 6 months to allow for an adjustment period following diagnosis,(3) met American College of Rheumatology (ACR)^13^ or SLICC (Systemic Lupus International Collaborating Clinics)^14^ classification criteria for SLE or had incipient SLE (‘Incipient SLE’ defined by 3 but < 4 ACR classification criteria for SLE). Patients who did not speak English fluently were excluded from the study.

### Study procedures

The study comprised two visits, the first of which involved the collection of socio-demographic data. During the second study visit,participants completed self-report depression and anxiety screening measures;parents (where available) completed a parent-about-child screening measure for anxiety symptoms only. Participants underwent a face-to-face semi-structured diagnostic psychiatric interview by a trained interviewer blinded to the self-report screening scores. Study visits were linked to routine outpatient SLE clinic visits. Participants who met diagnostic criteria for depressive or anxiety disorders following diagnostic interview were offered referral to the Department of Psychiatry for further assessment and/or treatment.Community volunteer hours (a requirement for high school graduation in Ontario) were provided to participants in appreciation for their time in study participation. As there is no age of consent in Canada,^15^ children and adolescents were deemed capable of ethically and medically consenting for their participation in the research presented in this manuscript. The study was approved by the Research Ethics Board of the Hospital for Sick Children (REB number 1000056640).

### Measures

#### CES-DC

The Center for Epidemiologic Studies Depression Scale for Children (CES-DC) is a freely available, widely-used, 20-item self-report screening questionnaire measure of current depressive symptoms among healthy and unwell children and adolescents aged 8-17 years.^16,17^ Convergent validity of the CES-DC with other depression inventories for children and young people has been demonstrated.^17^ Items inquire about symptoms occurring over the pastweek. The CES-DC has a four-factor structure^18^: depressed affect (7 items), somatic complaints (7 items), positive affect (4 items), and interpersonal problems (2 items). Responses are based on a four-point Likert Scale from 0-3 (0= “not at all”, 1= “a little”, 2 = “some”, 3= “a lot”). Four of the items (the positive affect domain) are phrased positively e.g. “I was happy” and are thus scored in reverse order(3 = “not at all” 2 = “a little”, 1 = “some”, 0 = “a lot”).The total score range is 0-60 and higher total scores reflect a greater level of self-reported depressive symptoms. Initial validation of the CES-DCas a screening measure for Major Depressive Disorder has suggested the cut-point score of 15 or greater as suggestive of potentially clinically significant depressive symptoms among children and adolescents in the community.^16^

### The screen for child anxiety related disorders (SCARED)

The SCARED is a freely available 41-item validated screening questionnaire used for assessment of anxiety symptoms in children and adolescents aged 8-18 years.^19^ The two available measures: The Parent about Child version and the Child (self-report) versionwere offered to the participant and the caregiver for completion. For the self-report, items are phrased as statements which describe feelings the child may have experienced during the past 3 months. Responses are based on a 3-point Likert Scale from 0-2 (0= “not true” or “hardly ever true;”; 1= “somewhat true” or “sometimes true”; 2= “very true” or “often true”). The possible score range is from 0-82. Items are further classified into five domains:generalized anxiety (8 items), separation anxiety (8 items), social phobia (7 items), school phobia (4 items) and panic/somatic anxiety (13 items). A total score of 25 or greater indicates a positive screen for the presence of clinically significant anxiety symptoms in community-based samples.

### Quality of my life (QOML)

The QOML is a measure of both Quality of Life (QOL) and Health Related Quality of Life (HRQOL) and was developed in a paediatric rheumatology sample.^20^ It contains three items assessing current ratings of QOL, HRQOL and change in QOL since the previous clinic visit. For the first two items, respondents are asked to record ratings for QOL and HRQOL on a 100 mm visual analogue scale ranging from 0 (the worst) to 100 (the best).The third item is the completion of the statement “Since the last time I was here, my life is…”with a response chosen from a 5-point ordinal scale (much worse, worse, about the same, better, much better).

### Kiddie schedule for affective disorders and Schizophrenia present and lifetime version 5

#### (K-SADS-PL-5)

The K-SADS-PL-5 has been validated in numerous research and clinical settings and consists of an interview screening section and six disorder-specific supplements.^21,22^ The instrument allows for a diagnostic assessment in accordance with DSM- 5 criteria not only for Major Depressive Disorder (MDD) but for all mood disorders. The brief introductory interview component allows the parent (and child) to provide important information regarding medical and psychiatric history, significant life events and social/educational functioning. This contextual information together with the diagnostic screen interview allows the interviewer to determine the presence of a current/past mood disorder diagnosis and their severity. Interviews were conducted by a child and adolescent psychiatrist (MQ) and clinical psychologist (AD) trained in the use of the instrument in patients with cSLE. The present study focused on current (four-week prevalence) diagnoses of mood and anxiety disorders. The Depression section of the K-SADS-Present Episode Version (K-SADS-Dep-C)^23^ and Anxiety supplements of the K-SADS-PL-5 were used to determine the presence of syndromal level symptoms.

### Demographic and disease data

The patient chart was used to provide data regarding date of birth, sex, medical history and associated medical treatments at the time of interview. Dates for cSLE medication treatments and medication doses including corticosteroids and immunosuppressants were recorded. cSLE specific data collected from the Hospital for Sick Children Division of Rheumatology SLE database included: (a) date of SLE diagnosis and SLE manifestations (ACR and SLICC classification criteria) (b) central nervous system involvement (other than mood disorder or anxiety disorder) (c) renal involvement (d) presence of antiphospholipid antibodies (e) joint pain/arthritis.

### Data analysis

Descriptive statistics were used to summarize the sample characteristics (means, standard deviation, range). Frequency analysis determined the distribution of screening measure scores for CES-DC (including domains), SCARED-Child and SCARED-Parent measures. Spearman’s correlation co-efficient was used to investigate the relationship between the non-parametrically distributed dichotomous MDD diagnostic status with CES-DC total score, CES-DC domains and QOL. Pearson’s correlation co-efficient investigated the relationship between CES-DC total score and QOL with CES-DC domains. Similarly, the relationship between both SCARED measures and QOL was investigated. Analyses were completed using Addinsoft 2019 XLSTAT statistical analysis and data solution software.

Receiver operating characteristics (ROC) analysis, Hanley and McNeil method^24^ was used to evaluate the performance of the CES-DC and both SCARED screeners against diagnostic interview to accurately detect current cases of MDD and any anxiety disorder (by DSM-5 criteria). Cases were defined as participants with diagnostic interview confirmed MDD or anxiety disorder. The ROC curve expresses the true positive rate (sensitivity) as a function of the false positive rate (1-specificity) for a range of screening measure cut-points and evaluates the ability of each screening measure to discriminate cases from non-cases. The area under the ROC curve (AUC) was estimated to quantify this and evaluate the diagnostic performance of each screening measure. An AUC value of 0.5 represents discriminatory ability no better than chance whereas a value of 1 represents perfect discriminatory ability.

## Results

### Participants

**Figure 1.**
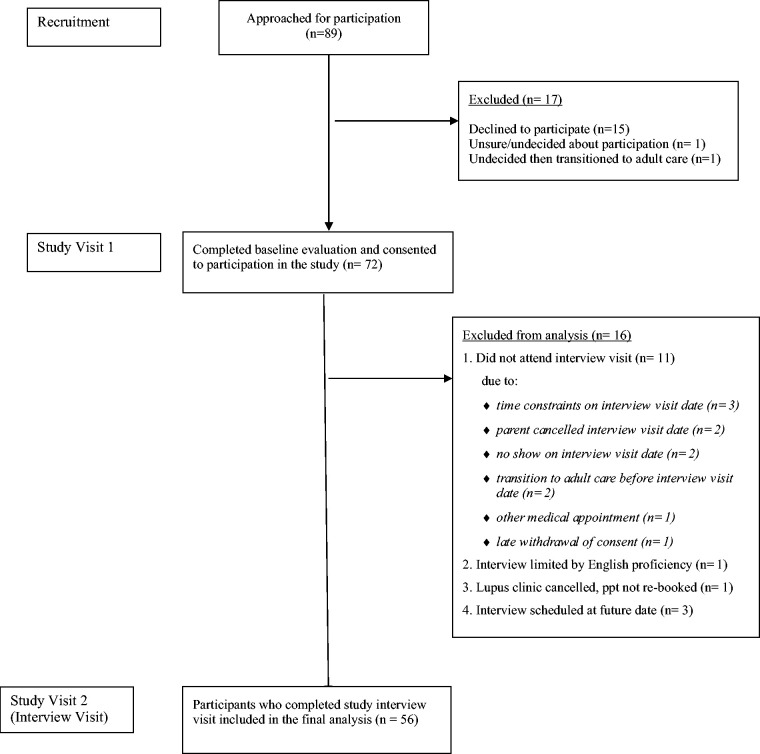
Flow diagram as per Strengthening the Reporting of Observational Studies in Epidemiology (STROBE) guidelines illustrating the enrolment of patients in the study.

Figure 1 shows the Strengthening the Reporting of Observational Studies in Epidemiology (STROBE)^25^ flow diagram of participant enrolment in the study. Of 89 patients who were approached, 72 children and adolescents (81%) consented to take part and completed baseline study questionnaires and demographic forms at the first study visit. Sixteen participants were excluded from the final analysis (see Figure 1). The study cohort consisted of 56 patients with SLE of which there were 50 complete parent/child pairs. Table 1 outlines the demographic and disease characteristics of thestudy participants. The mean participant age was 15.4 years (SD2.1 years, range 9-17 years); 84% were female. The most frequent racial/ethnic group was East Asian (26.7%) followed by South Asian, White/Caucasian (both 19.7%) and Hispanic (17.9%).

**Table 1.** Demographic and disease characteristics of participants (N = 56)

Baseline characteristics	N (%)
Age (years)	
Mean (SD)	15.4 (2.1)
Range	9–17
Female, *N* (%)	47 (83.9)
Race/Ethnicity, *N* (%)	
White/Caucasian	11 (19.7)
Black/African Canadian	2 (3.5)
East Asian	15 (26.7)
South Asian	11 (19.7)
Hispanic	10 (17.9)
Mixed race	4 (7.1)
Other	3 (5.3)
Diagnosis: cSLE, *N* (%)	51 (89.2)
Incipient cSLE, *N* (%)	5 (10.8)
SLE manifestations (ever) *N (%)*	
Arthritis	3 (5.3)
Renal disease	22 (39.4)
History of joint pain	8 (14.2)
Median SLEDAI score	0.55
Mean Disease duration (years)	4.3
(SD, Range)	(2.7, 11.6)
Current corticocosteroid use (prednisone equivalent)	
Low dose (1-10mg/day)	12 (21.4)
High dose (>10mg/day)	4 (7.1)
Current antidepressant medication use, *N (%)*	2 (3.5 )
NPSLE manifestations (ever) *N (%)*	
Any NPSLE manifestation,	7 (12.5)
Psychosis	2
Headache	2
Acute confusional state	1
Seizures	1
Chorea	1
Prior psychiatric assessment, *N (%)*	4 (7.1)
Positive psychiatric history in first degree relative, *N (%)*	16 (28.5)

### Screening measures

The mean CES-DC score of the sample (n = 56) was 14.9 (SD12, range1- 49) (Table 2). The CES-DC screen positive rate for the sample was 35.7%. The frequency distribution of the CES-DC, SCARED-Child and SCARED-Parent screening measure scores among cSLE respondents are represented in Supplemental Figure 1. CES-DC domains were moderate to strongly associated with total CES-DC scores: somatic complaints (r = 0.92), depressed affect (r = 0.95), (lack of) positive affect (r = 0.59), interpersonal problems (r = 0.71) (p < 0.05) (see Supplemental Table 1).

**Table 2.** Prevalence of reported depressive and anxiety symptoms in cSLE study participants using the CES-DC, SCARED (Child) and SCARED (Parent) Measures.

	CES-DC (total) (N = 56)	CES-DC (Som)^a^	CES-DC (DA)^b^	CES-DC (lack of PA)^c^	CES-DC (IP)^d^	SCARED- Child (N = 56)	SCARED-Parent (N = 50)*
Mean score (SD)	14.9 (12)	5.3 (4.9)	4.1 (4.9)	4.6 (2.6)	0.9 (1.5)	22 (14)	13 (8)
Range	1–49	0–21	0–18	0–11	0–6	2–61	0–36
Positive screen N (%)	20 (35.7)	N/A	N/A	N/A	N/A	22 (39.2)	5 (10)

*Missing observations (n = 6).

^a^Som: somatic symptoms.

^2^DA: depressed affect.

^3^Lack of PA: lack of positive affect.

CES-DC screen positive if total score >15, SCARED-Child screen positive if total score >25, SCARED-Parent screen positive if total score > 25.

N/A: not applicable.

The mean SCARED-Child screening score (n = 56) was 22 (SD = 14, range= 2-61) (Table 2). Based on completed screening measures available (n = 50), the mean SCARED-Parent screening score of the sample was 13 (SD 8, range 0-36). Screen-positivity for the SCARED-Child and SCARED-Parent reports were 39.2% and 10% respectively. The SCARED-Child and SCARED-Parent measure scores had a similarly weak correlation with anxiety disorder diagnosis (r = 0.27 and r = 0.25 respectively) (Supplemental Table 1). Correlation between SCARED-Child and the corresponding SCARED-Parent screening measure score was weak (r = 0.33).

The mean QOL score for the sample was 70 (SD16.6, range 30-100). Mean HRQOL score was 64 (SD20.2, range 21-98) (missing observations n = 5). CES-DC total score had a moderate negative correlation with overall QOL (r = -0.57) and with HRQOL (r = -0.44). SCARED-Child measure correlated weakly with QOL and HRQOL (r = -0.28 and r = -0.36 respectively) (see Supplemental Table 2). SCARED-Parent measure showed the weakest relationships with QOL (r = -0.08) and HRQOL (r = -0.04) (see Supplemental Table 2).

### K-SADS DSM-5 psychiatric diagnoses

Current and past psychiatric disorder by diagnostic interview are summarized by DSM-5 diagnosis in Table 3. The prevalence of current MDD was 5.3% (n = 3). No participant met criteria for diagnosis of persistent depressive disorder (dysthymia/chronic depression). One participant (1.7%) was diagnosed with medication-induced depressive disorder. With respect to any previous history of mood disorder, 6 participants (10.7%) met criteria for previous MDD. Four (7.1%) participants met criteria for a past diagnosis of medication (corticosteroid) induced depressive disorder. One participant endorsed a past adjustment disorder with depressed mood and one participant endorsed a past mood disorder due to medical condition (cSLE).

**Table 3.** Prevalence of DSM-5 psychiatric diagnoses in cSLE study participants by K-SADS interview (n = 56).

Current psychiatric disorder	N (%)
Major Depressive Disorder	3 (5.3)
Medication (corticosteroid) induced depressive disorder	1 (1.7)
Persistent Depressive Disorder	0
Anxiety Disorders (all)	9 (16.1)
Social Anxiety Disorder	4 (7.1)
Generalized Anxiety Disorder	3 (5.3)
Panic Disorder	1 (1.7)
Specific Phobia	1 (1.7)
Obsessive Compulsive Disorder	0
Past Psychiatric Disorder	
Previous Major Depressive Disorder	6 (10.7)
Medication (corticosteroid) induced depressive disorder	4 (7.1)
Attention Deficit Hyperactivity Disorder*	2 (3.5)
Adjustment disorder with depressed mood	1 (1.7)
Mood disorder due to medical condition (cSLE)	1 (1.7)
Anorexia Nervosa*	1 (1.7)

*Diagnoses reported in past psychiatric history during interview and verified in the patient chart.

The prevalence of any current anxiety disorder was 16.1% (n = 9). Social anxiety disorder was the most prevalent anxiety disorder (7.1%), followed by generalized anxiety disorder (5.3%). Other anxiety disorders are provided in Table 3. The K-SADS interview also provided other previous psychiatric diagnoses of Attention Deficit Hyperactivity Disorder (n = 2; 3.5%) and Anorexia Nervosa (n = 1; 1.7%).

### ROC and sensitivity and specificity analyses

**Figure 2.**
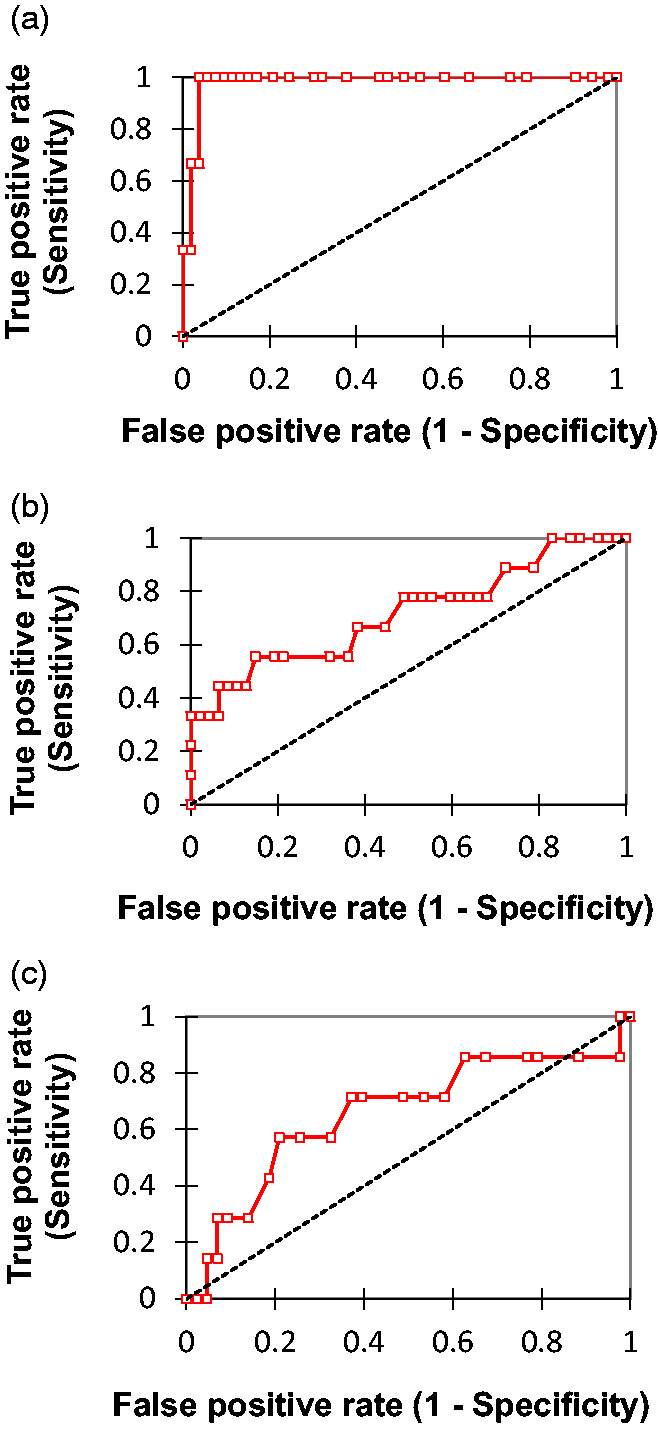
Receiver Operating Characteristics (ROC) curves for the detection of depressive and anxiety disorders using the CES-DC and SCARED (Parent) and SCARED (Child) measures.

The ability of each screening measure to correctly identify patients with mental disorders from the corresponding diagnostic groups is reported in Figure 2 by calculation of the ROC Area under the Curve (ROCAUC). Accompanying sensitivity, specificity, positive and negative predictive values (PPV and NPV, respectively) over a range of scores for the study screening measures, are provided with optimal cut-points in Table 4.

**Table 4.** Sensitivity, specificity, positive and negative predictive values for a range of cut-points using CES-DC, SCARED-Child and SCARED-Parent screening measures.

Score	Sensitivity	Specificity	PPV	NPV	TP	TN	FP	FN
CES-DC							
15	1	0.7	0.15	1	3	37	16	0
20	1	0.83	0.24	1	3	44	9	0
25	1	0.92	0.41	1	3	49	4	0
30	1	0.94	0.48	1	3	50	3	0
35	1	0.96	0.58	1	3	51	2	0
38	0.67	0.96	0.48	0.98	2	51	2	1
SCARED-							
Child score							
20	0.78	0.51	0.23	0.92	7	24	23	2
25	0.56	0.68	0.25	0.89	5	32	15	4
30	0.56	0.85	0.42	0.91	5	40	7	4
32	0.44	0.87	0.44	0.89	4	41	6	5
SCARED-							
Parent score							
15	0.57	0.67	0.25	0.89	4	29	14	3
17	0.57	0.74	0.3	0.9	4	32	11	3
18	0.57	0.79	0.34	0.91	4	34	9	3
20	0.29	0.86	0.28	0.86	2	37	6	5
26	0.14	0.93	0.28	0.85	1	40	3	6

The CES-DC achieved an AUC of 0.98 in detecting participants with Major Depressive Disorder (*p* < 0.01). The optimal cut-point was a CES-DC score of 35 (point of maximum sensitivity+ specificity). Sensitivity of the CES-DC at this score was 100% and specificity was 96% with positive predictive value of 58% and a negative predictive value of 100%.The SCARED-Child measure ROCAUC was 0.71 (*p* < 0.05). The optimal cut-point of 30 gave a sensitivity of 56% and a specificity of 85% for this screening measure with PPV of 42% and NPV of 91%. The SCARED-Parent measure ROCAUC was 0.66 however *p*value did not reach statistical significance. The optimal cut-point was 18 providing a sensitivity of 0.57 and specificity of 0.79. PPV and NPV values at this cut-point were 0.34 and 0.91 respectively.

## Discussion

This study reports on the self-report screening measure and standardized diagnostic interview outcomes for 56 children and adolescents with cSLE. We found that both the CES-DC and the SCARED-Child screening measures significantly overestimated the prevalence of MDD and anxiety disorder, respectively, confirmed by diagnostic psychiatric interview. The prevalence rate for symptom screen-positivity by self-report measure compared to gold standard diagnostic interview was 35.7% versus 5.3% for MDD on the CES-DC and 39% versus 16% for anxiety disorder the SCARED-Child. Depressive and anxiety disorder prevalence rates in our cSLE study sample are similar to those in healthy community samples of children and adolescents.^26,27^

Several factors may account for the discrepant prevalence rates of the screening measure and diagnostic interview. Of significance is the difference in measurement approach - the dimensional nature of the CES-DC measure does not closely map symptom screen outcomes on to a DSM-5 categorical diagnosis of mental disorder.

Self-report measures may under or over-report symptoms of anxiety and depression for many reasons including state-mediated effects of psychopathology on reporting, cognitive impairment due to illness and treatments or fear of stigmatizing attitudes from clinicians and/or caregivers.^9,28^ These measures are highly sensitive to adjustment difficulties and psychological distress which can trigger adolescent mood fluctuations and emotional reactivity. This finding replicates other studies of depression in chronically ill paediatric patients in which screening measures have been shown to be highly sensitive to psychological distress and yet less effective in discriminating psychiatrically ill children.^29^ This underlines the challenges of screening for depressive disorder in children and youth with chronic disease.

### Screening measure cut-points

The best screening measure performance was observed for the CES-DC measure with an AUC of 0.98 by ROC-analysis. Optimal cut-points were high for both screening measures in our cSLE sample relative to the cut-point thresholds for screen positivity in community samples. Our study determined an optimal cut-pointof 35 for the CES-DC when used in cSLE, with a sensitivity of 100% and specificity of 96%. This suggeststhat in cSLE, considerably higher CES-DC scores than the community sample cut-point of 15 maybe required to discriminate between cSLE patients with MDD and those without the disorder.

Notwithstanding the elevation of CES-DC measure scores through psychological distress, the relative contribution of somatic symptoms may also be significant. In our sample, high scores in the somatic domain of the CES-DC (e.g. fatigue, poor concentration) may reflect true depressive symptoms,may represent active SLE which is commonly associated with fatigue, or may be a side-effect of medical treatment. When combined, these factors may serve to explain the high number of false positive screening outcomes for MDD when the CES-DC screen-positive threshold is set at the community cut-point of 15. Children with SLE may already have higher baseline somatic items attributable to their disease and the presence of co-morbid depressive illness may serve to inflate somatic symptoms significantly. Based on examination of lower CES-DC cut-points, an argument could be made for a cut-point of 25, also the cut-point for psychiatric populations,^30^ as appropriate due to minimal impact on sensitivity and specificity (100% and 92% respectively). The “cost” outlined in Table 4 in the balance of false positive and false negative screens is an increase from 2 to 4 of false positive screens.

Treating the CES-DC somatic symptom domain with caution is important in medically ill children who may have a propensity for falsely elevated scores and the potential for a false positive diagnosis. However, the disease activity index of the study participants was noted to be low which may mean that CES-DC elicited true somatic depressive symptoms for many participants despite this situation of overlap. The inclusion of somatic symptoms questions in the CES-DC could be viewed as a strength of this measure given that children, and particularly medically ill children, may be more likely to report somatic depressive symptoms. Moreover, when somatic symptoms accompany cognitive and emotional/behavioural symptoms, the course of illness may be more severe and such children may have poorer outcomes.^31,32^

The SCARED offers the opportunity for the parent-report measure to complement the self-report measure, however, most studies show low to moderate agreement between parent and child reported anxiety symptoms.^33^ Unfortunately, ROC-analysis of SCARED parent report measures was limited by missing parent-report data, and the lack of statistical significance of the result did not allow for a direct comparison of diagnostic performances of SCARED parent and self-report measures in cSLE. Correlation(r = 0.33) between parent and self-report SCARED measures suggested weak agreement between parent and child informants as seen in other studies of healthy children,^34^ in which children have repeatedly been found to be more accurate informants of their anxiety than their parents.^35^

### Presence of subthreshold versus threshold symptoms

The DSM-5 diagnostic criteria for MDD in children and youth specify the pervasive presence of five of nine depressive symptoms, for two weeks or more with a clinically significant impairment in functioning. Symptoms directly arising from the SLE disease process (or medical treatment) do not qualify for a comorbid MDD diagnosis. The diagnosis of anxiety disorder by DSM-5 follows a similarly strict set of diagnostic criteria. As such, standardized psychiatric interview captures a distinct subset of children and adolescents with more severe symptoms and impairment, whereas screening measures may capture milder psychological distress or measure other symptoms of disease or associated treatment.

Despite the low prevalence rate of 5.3% for MDD in our sample, the high CES-DC screen positive rate of 35.7% may represent a high point prevalence of subthreshold depressive symptoms. It highlights the existence of a subgroup of “stressed, but not depressed” children in our cSLE sample who may progress to diagnostic threshold symptom severity. Such prolonged stress in susceptible individuals may result in hypothalamic-pituitary adrenal axis activation and set forth a pro-inflammatory cytokine cascade.^36^ Vulnerability to this stress-induced pro-inflammatory state may be a risk factor in cSLE for the progression of subthreshold depressive symptoms to MDD. In screen-positive clinical encounters pediatric rheumatology clinicians should explore with the patient and their caregiver any inconsistencies between self-report questionnaires and the clinical presentation using a biopsychosocial approach. A lower threshold for seeking a psychiatric assessment may be appropriate for children and adolescents with persistent psychological distress and in particular those with significant risk factors for mental disorder. Where self-report measures are elevated, and in the presence of risk factors and/or clinician index of suspicion of mental disorder, ideally, follow-up with a mental health professional (child and adolescent psychiatrist, clinical psychologist) would take place.

### Study strengths and limitations

The main strength of the present study is the use of semi-structured, diagnostic psychiatric interview as the gold standard against which the diagnostic performance of screening measures is measured. To our knowledge, these are the first robust prevalence data on the co-morbidity of depressive and anxiety disorders with cSLE. The representativeness of the study sample within the Greater Toronto Area with respect to race/ethnicity increases the generalizeability of the study results.This study replicates, with a larger sample size, the findings from a previous study of depressive symptoms in cSLE^37^ and shows mean depressive screening scores just below threshold for screen positivity. The provision of psychiatric diagnosis allows for meaningful longitudinal studies to take place regarding prevalence and correlates of depressive and anxiety disorders in cSLE.

Although the current study is the largest of its kind to date, the low prevalence rate of MDD and anxiety disorders resulted in limited data regarding screening measure outcomes on which to assess diagnostic performance. While previous studies examining depression screening outcomes in cSLE have had higher disease activity compared to the present study, these studies have not had depressive disorder diagnoses confirmed by gold standard interview.^37,38^ The cross-sectional nature of the study and convenience sample with relatively stable disease may have precluded sample enrichment for specific disease symptoms or specific disease timepoints. Data regarding depressive and anxiety disorders in younger children, and boys in particular, are lacking due to the preponderance of adolescent females in the study. Future studies of larger sample sizes, enriched for psychological distress, are needed to examine the generalizeability of our findings to boys and to younger children with cSLE.

## Conclusions

This study finds a similar prevalence rate of major depressive disorder and anxiety disorders among children with SLE as in community samples.^39^ These preliminary findings underline the importance of examining the validity of psychiatric screening measures in special populations such as children with SLE prior to implementation. Pediatric rheumatology clinics screening for depressive and anxiety disorders are likely to find high rates of distress, among which disorder may be present.

## Supplemental Material

sj-pdf-1-lup-10.1177_09612033211018504 - Supplemental material for Evaluation of self-report screening measures in the detection of depressive and anxiety disorders among children and adolescents with systemic lupus erythematosus Click here for additional data file. Supplemental material, sj-pdf-1-lup-10.1177_09612033211018504 for Evaluation of self-report screening measures in the detection of depressive and anxiety disorders among children and adolescents with systemic lupus erythematosus by Michelle Quilter, Linda Hiraki, Andrea M Knight, Julie Couture, Deborah Levy, Earl D Silverman, Ashley N Danguecan, Lawrence Ng, Daniela Dominguez, Katherine T Cost, Kate M Neufeld, Reva Schachter and Daphne J Korczak in Lupus

sj-pdf-2-lup-10.1177_09612033211018504 - Supplemental material for Evaluation of self-report screening measures in the detection of depressive and anxiety disorders among children and adolescents with systemic lupus erythematosus Click here for additional data file. Supplemental material, sj-pdf-2-lup-10.1177_09612033211018504 for Evaluation of self-report screening measures in the detection of depressive and anxiety disorders among children and adolescents with systemic lupus erythematosus by Michelle Quilter, Linda Hiraki, Andrea M Knight, Julie Couture, Deborah Levy, Earl D Silverman, Ashley N Danguecan, Lawrence Ng, Daniela Dominguez, Katherine T Cost, Kate M Neufeld, Reva Schachter and Daphne J Korczak in Lupus

sj-pdf-3-lup-10.1177_09612033211018504 - Supplemental material for Evaluation of self-report screening measures in the detection of depressive and anxiety disorders among children and adolescents with systemic lupus erythematosus Click here for additional data file. Supplemental material, sj-pdf-3-lup-10.1177_09612033211018504 for Evaluation of self-report screening measures in the detection of depressive and anxiety disorders among children and adolescents with systemic lupus erythematosus by Michelle Quilter, Linda Hiraki, Andrea M Knight, Julie Couture, Deborah Levy, Earl D Silverman, Ashley N Danguecan, Lawrence Ng, Daniela Dominguez, Katherine T Cost, Kate M Neufeld, Reva Schachter and Daphne J Korczak in Lupus
