# Supplementary material for: Evaluation of self-report screening measures in the detection of depressive and anxiety disorders among children and adolescents with systemic lupus erythematosus
Source: Lupus. 2021 Jun 2;30(8):1327–37. doi: 10.1177/09612033211018504 (PMC8209764; doi:10.1177/09612033211018504)
Supplement: sj-pdf-1-lup-10.1177_09612033211018504 - Supplemental material for Evaluation of self-report screening measures in the detection of depressive and anxiety disorders among children and adolescents with systemic lupus erythematosus [file sj-pdf-1-lup-10.1177_09612033211018504.pdf]

Supplemental Table 2. Pearson correlation of QOL and HRQOL with SCARED- Child and SCARED- Parent, ($p < 0.05$)

| | | SCARED- Child | SCARED- Parent |
|---|---|---|---|
| Pearson’s r | QOL | -0.28 | -0.08 |
| | HRQOL | -0.36 | -0.04 |
