# Supplementary material for: Evaluation of self-report screening measures in the detection of depressive and anxiety disorders among children and adolescents with systemic lupus erythematosus
Source: Lupus. 2021 Jun 2;30(8):1327–37. doi: 10.1177/09612033211018504 (PMC8209764; doi:10.1177/09612033211018504)
Supplement: sj-pdf-2-lup-10.1177_09612033211018504 - Supplemental material for Evaluation of self-report screening measures in the detection of depressive and anxiety disorders among children and adolescents with systemic lupus erythematosus [file sj-pdf-2-lup-10.1177_09612033211018504.pdf]

Supplemental Table 1. Spearman correlation of diagnosis MDD with CES-DC total score, CES-DC domains and QOL (*[illegible]*

		CES-DC total (N=56)	CES-DC (Som)[1]	CES-DC (DA)2	CES-DC (lack of PA)3	CES-DC (IP)4
Spearman's r	DSM-5 MDD	0.37	0.38	0.37	0.21*	0.38
Pearson's r	CES-DC total	1	0.92	0.95	0.59	0.71
	QOL	-0.57	-0.52	-0.49	-0.38	-0.36
	HRQOL	-0.44	-0.48	-0.33	-0.20*	-0.33

[1] Som = somatic symptoms, [2]DA = depressed affect, [3]lack of PA = lack of positive affect, [4]IP = interpersonal problems,

[illegible] < 0.05); Pearson correlation of CES-DC total score and QOL with CES-DC domains ($p < 0.05$)

QOL (N= 51)	HRQOL (N=51)
-0.26*	-0.27*
-0.57	-0.44
1	NR
NR	1

NR = not recorded, *$p > 0.05$
