# Supplementary material for: Evaluation of self-report screening measures in the detection of depressive and anxiety disorders among children and adolescents with systemic lupus erythematosus
Source: Lupus. 2021 Jun 2;30(8):1327–37. doi: 10.1177/09612033211018504 (PMC8209764; doi:10.1177/09612033211018504)

Supplemental Figure 1. Frequency distribution of the CES-DC, SCARED-Child and SCARED-Parent screening measure scores among cSLE respondents

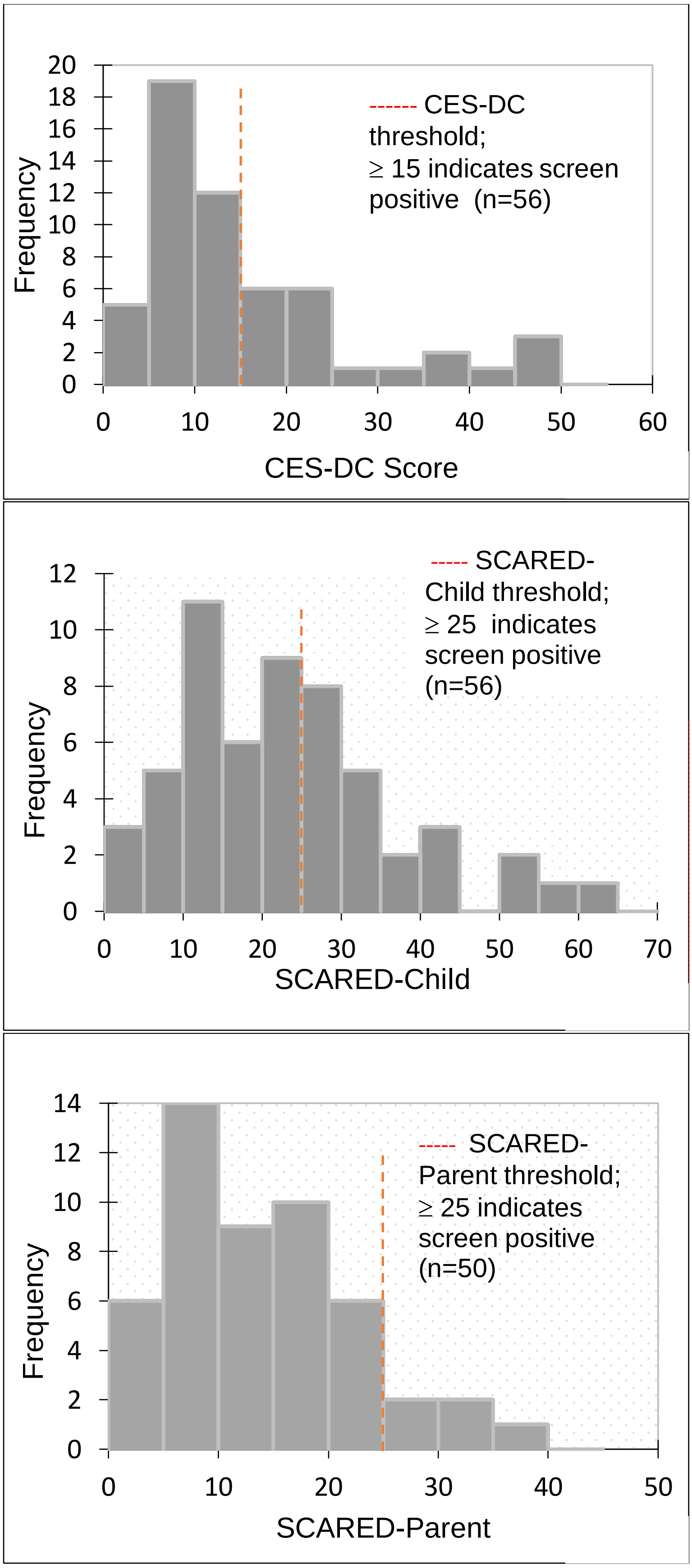

Supplement: sj-pdf-3-lup-10.1177_09612033211018504 - Supplemental material for Evaluation of self-report screening measures in the detection of depressive and anxiety disorders among children and adolescents with systemic lupus erythematosus [file sj-pdf-3-lup-10.1177_09612033211018504.pdf]
